# Strategies for combating persister cell and biofilm infections

**DOI:** 10.1111/1751-7915.12774

**Published:** 2017-07-11

**Authors:** Thomas K. Wood

**Affiliations:** ^1^ Department of Chemical Engineering Pennsylvania State University University Park Pennsylvania 16802‐4400 PA USA

## Abstract

Bacterial cells are constantly exposed to environmental stress; for example, almost all cells must endure starvation, and antimicrobials, of course, are administered to kill bacteria. These stressed cells enter a resting state known as persistence in which they become tolerant to nearly all antibiotics without undergoing genetic change. These dormant cells survive courses of antibiotics, as antibiotics are most effective against actively metabolizing cells, and reconstitute infections. In humans, most of these bacterial infections occur in biofilms in which bacteria attach to one another via secreted proteins, polysaccharides and even DNA. Herein, biotechnological methods are described to combat persister cells and to eradicate biofilms by understanding the genetic basis of both phenomena.

## Abstract 2

Bacterial cells are constantly exposed to environmental stress; for example, almost all cells must endure starvation, and antimicrobials, of course, are administered to kill bacteria. These stressed cells enter a resting state known as persistence in which they become tolerant to nearly all antibiotics without undergoing genetic change. These dormant cells survive courses of antibiotics, since antibiotics are most effective against actively‐metabolizing cells, and reconstitute infections. In humans, most of these bacterial infections occur in biofilms in which bacteria attach to one another via secreted proteins, polysaccharides, and even DNA. Herein, biotechnological methods are described to combat persister cells and to eradicate biofilms by understanding the genetic basis of both phenomena.

## Sustainable development goal and scope

As bacteria evolve resistance to all antimicrobials and even compounds that prevent them from communicating (Maeda *et al*., 2012), the goal is to develop new, sustainable techniques for treating bacterial infections by understanding how persister cells and biofilms form. Furthermore, the cost of all biofilm/persister infections to society is substantial; for example, 17 million new biofilm infections occur every year in the United States, and of these infections, 550 000 people die (Wolcott and Dowd, 2011). In addition, biofilm infections add more than $1B to the cost of hospital stays (Percival *et al*., 2011) as bacterial infections have been found for most if not all medical devices (Bryers, 2008) and surgical removal is the only recourse. Furthermore, 1–2% of those in developed countries will develop chronic skin wounds, which cost $25B annually in the United States alone (Percival *et al*., 2011).

## Combating persister cells

Persister cells survive the stress of antibiotic treatment due to their lack of metabolism, rather than through genetic change, as shown via four seminal experiments conducted by the discoverers of the phenotype (Hobby *et al*., 1942; Bigger, 1944); later, once the antibiotic is removed, the cells can reconstitute infections. Subsequent research corroborated that persister cells are metabolically inactive; for example, Shah *et al*. (2006) found that metabolically inactive cells were more tolerant to the fluoroquinolone ofloxacin, and Kwan *et al*. (2013) found that cells lacking protein synthesis become persister cells, via pretreatment with rifampicin to stop transcription, with tetracycline to stop translation or with carbonyl cyanide *m*‐chlorophenylhydrazone to halt ATP production. These three pretreatments convert an initial population of 0.01% persisters to up to approximately 80% persisters (a 10 000‐fold increase in persister cells). Recent evidence has confirmed the importance of reducing protein production in persistence by demonstrating that the persister cells have sharply reduced ATP levels (Conlon *et al*., 2016). Hence, persister cells are predominantly dormant.

As persister cells are dormant and resistant to traditional antibiotics (e.g. fluoroquinolones, aminoglycosides and β‐lactams), microbial biotechnological approaches have been developed to kill sleeping cells. These approaches must utilize compounds that enter the cell without the need of active transport and kill the persister cells without requiring any cell machinery (as there is little or no metabolism). Examples of this approach include utilizing the DNA‐cross‐linking agents mitomycin C (Kwan *et al*., 2015) and cisplatin (Chowdhury *et al*., 2016); both compounds are approved for human use as cancer treatments by the U.S. Food and Drug Administration (FDA) and hold great promise for treating persistent infections, such as those related to wounds, because they have been shown to be effective for a wide range of infections including those of commensal *E. coli* K‐12 as well as the pathogenic species *E. coli* O157:H7 (EHEC), *S. aureus* and *P. aeruginosa*. Another example of killing persister cells as they sleep is based on tricking ClpP protease to degrade many cellular proteins by adding the acyldepsipeptide ADEP4 (Conlon *et al*., 2013); this approach was successful with *S. aureus* infections in a mouse model when ADEP4 is combined with other antibiotics like rifampicin (Conlon *et al*., 2013).

An alternative approach is to wake persister cells and then treat them with traditional antibiotics because adding sugars and glycolysis intermediates (e.g. mannitol, glucose, fructose, pyruvate) rapidly wakes persister cells (Allison *et al*., 2011). Similarly, *P. aeruginosa* persister cells may also be awakened with *cis*‐2‐decenoic acid, which causes a burst in protein synthesis, and then killed by ciprofloxacin (Marques *et al*., 2014).

As with many biotechnological approaches, magic bullets for combating persister infections are rare. Far more likely is that a combination of compounds will be necessary to effectively treat persistent infections as was done recently for treating Lyme disease; by combining three antibiotics, the lipopeptide daptomycin, the beta‐lactam cefoperazone and tetracycline‐class doxycycline, an effective cocktail was made for combating infections by *Borrelia burgdorferi* (Feng *et al*., 2015).

## Combating biofilm infections

Biofilms are the homes of bacteria in which they can better weather stress; these homes consist of a dense extracellular matrix that cements cells together. This matrix usually is composed of exopolysaccharides, extracellular DNA and proteins (Whitchurch *et al*., 2002; Branda *et al*., 2005; Franklin *et al*., 2011; Lister and Horswill, 2014; Fong and Yildiz, 2015). During times of both feast and famine (Kaplan, 2010), bacteria frequently degrade their own biofilms so they may colonize other areas (Karatan and Watnick, 2009); this requires secreting enzymes and is known as biofilm dispersal. Hence, an exciting, new, microbial biotechnological approach to remove biofilms is to induce their own cellular machinery to remove their biofilms. For example, as the biofilm matrix *P. aeruginosa* biofilm consists of alginate, Pel polysaccharide, Psl polysaccharide (Franklin *et al*., 2011) and extracellular DNA (Whitchurch *et al*., 2002; Jennings *et al*., 2015), this organism produces the glycoside hydrolase PelA to remove its Pel polysaccharide (Baker *et al*., 2016) and the glycoside hydrolase PslG to remove its Psl polysaccharide (Yu *et al*., 2015). Similarly, *Actinobacillus actinomycetemcomitans* produces the glycoside hydrolase dispersin B to degrade the *N*‐acetyl β‐*D*‐glucosamine (GlcNAc) in its own matrix (Ramasubbu *et al*., 2005); because GlcNAc is also part of the matrix *Staphylococcus epidermidis*,* Escherichia coli*,* Yersinia pestis* and *P. fluorescens* biofilms, dispersin B can degrade these biofilms as well (Itoh *et al*., 2005). Showing the promise of this biotechnological approach, DNase is in clinical use for disrupting *P. aeruginosa* biofilms and dispersin B is also a possible therapeutic enzyme (Baker *et al*., 2016).

As persister cells frequently arise in biofilms (Lewis, 2008), it is important to treat both persister cells in suspension and within biofilms; this has been shown to be possible with compounds like *cis*‐decenoic acid, which causes a 3000‐fold reduction in the persister cells of the opportunistic pathogen *P. aeruginosa* in planktonic cultures along with a million‐fold reduction in biofilm‐derived persisters (Marques *et al*., 2014). Similarly, mitomycin C eliminates pathogenic *E. coli* and *S. aureus* in both suspension and biofilms (Kwan *et al*., 2015), and cisplatin eradicates *P. aeruginosa* persister cells in both biofilms and suspension (Chowdhury *et al*., 2016). Furthermore, some compounds have been discovered that both remove biofilms as well as kill persisters; for example, halogenated phenazines remove biofilms of *S. aureus* as well as kill its persister cells (Garrison *et al*., 2015).

The main challenge for these biotechnological discoveries is translating these laboratory developments into clinical use. To date, only a handful of antibiofilm compounds have been shown to be efficacious with humans. For example, 5‐fluorouracil was utilized successfully in a human trial (Walz *et al*., 2010) and was given FDA approval for use to prevent biofilm formation on catheters (Angiotech Pharmaceuticals); 5‐fluorouracil was discovered by screening 6,000 *P. aeruginosa* mutants for changes in biofilm formation and works by reducing cell communication (Ueda *et al*., 2009). 5‐Fluorouracil was initially an FDA‐approved for treating cancer (like mitomycin C and cisplatin), which illustrates another promising approach: repurposing drugs for antipersister and antibiofilm use (Soo *et al*., 2017). Therefore, given these exciting discoveries for treating the most recalcitrant infections, one can be sanguine about our ability to continue to make use of biotechnology for combating infections.

## Conflict of interest

None declared.
